# The role of plant growth promoting rhizobacteria in plant drought stress responses

**DOI:** 10.1186/s12870-023-04403-8

**Published:** 2023-08-25

**Authors:** Maha Chieb, Emma W. Gachomo

**Affiliations:** Department of Microbiology and Plant Pathology, University of California Riverside, Riverside, CA 92507 USA

**Keywords:** Beneficial bacteria, PGPR, Plant growth, Abiotic stress, Phytohormones, Drought stress tolerance

## Abstract

Climate change has exacerbated the effects of abiotic stresses on plant growth and productivity. Drought is one of the most important abiotic stress factors that interfere with plant growth and development. Plant selection and breeding as well as genetic engineering methods used to improve crop drought tolerance are expensive and time consuming. Plants use a myriad of adaptative mechanisms to cope with the adverse effects of drought stress including the association with beneficial microorganisms such as plant growth promoting rhizobacteria (PGPR). Inoculation of plant roots with different PGPR species has been shown to promote drought tolerance through a variety of interconnected physiological, biochemical, molecular, nutritional, metabolic, and cellular processes, which include enhanced plant growth, root elongation, phytohormone production or inhibition, and production of volatile organic compounds. Therefore, plant colonization by PGPR is an eco-friendly agricultural method to improve plant growth and productivity. Notably, the processes regulated and enhanced by PGPR can promote plant growth as well as enhance drought tolerance. This review addresses the current knowledge on how drought stress affects plant growth and development and describes how PGPR can trigger plant drought stress responses at the physiological, morphological, and molecular levels.

## Highlight

This review aims to highlight the recent advances in understanding the effects of plant growth-promoting rhizobacteria in enhancing plant growth and drought stress tolerance.

## Background

Drought is one of the major abiotic stress factors that hinders plant growth and development, limits agricultural productivity, significantly increases the cost of agricultural production, and ultimately impacts the food and feed supply. Plants use a variety of interconnected physiological, biochemical, molecular, nutritional, metabolic, and cellular mechanisms to respond to drought stress. Research has demonstrated that inoculating plants with plant-growth-promoting rhizobacteria can be an effective environmentally friendly strategy to foster plant growth and mitigate the negative effects of drought. In this review, we highlight current advances in understanding the role of PGPR in inducing plant adaptations to drought stress and in promoting plant growth. We outline the direct and indirect mechanisms used by the PGPR to positively impact plant development under drought stress.

## Introduction

Over the last two decades, climate change has become one of the most important environmental concerns. As a results of global warming, the impacts of abiotic stress factors such as temperature shifts and extremes, increase in atmospheric carbon dioxide, excessive light, floods, drought, salinity, alkaline and acidic soils, heavy metal pollution, toxic and hazardous chemicals, and nutrient deficient soils on plant growth have increased [1]. Climate change has a significant influence on the Earth’s water cycle and the rising temperatures increase atmospheric water evaporation rates, reducing water availability and drying off soils and vegetation [2]. This leads to more frequent and intense rainstorms that cause flooding in some areas, while other areas experience surface water deficit, and dry soils resulting in prolonged drought. Water shortage and rising aridity often coincide with high temperatures; drought is one of the most prevalent and complex abiotic stress factors for plants. Dry soils and vegetation negatively affect agricultural production, which cause food shortages and increase forest fires [3]. During drought, stomata gradually close with a concurrent decline in water retention, thus adversely affecting water use efficiency, plant diffusion, nutrient absorption, and transport from roots to shoots, which alter plant physiological, biochemical, morphological, and molecular traits [4]. Indeed, drought stress hampers the major cellular processes that determine plant growth and productivity such as cell division, elongation, and differentiation which consequently affect plant growth factors including root proliferation, leaf size, and stem extension [3]. Moreover, a reduction in stomatal and mesophyll conductance due to drought stress restricts carbon dioxide (CO_2_) diffusion, photorespiration, energy, membrane integrity, pigment content, lipid and cellular elements biosynthesis, and hormone balance [5, 6]. Besides the complexity of drought effects themselves, the regulatory mechanisms for drought tolerance in plants are complex, and require many biological responses, from signaling to drought stress resistance. Thus, plants may adopt several relative resistance responses to maintain their functional growth in order to alleviate the detrimental effects of drought stress [3].

Several drought management strategies for agricultural fields might be beneficial in reducing the impact of drought on crop production and yield. For example, the classical selection approaches and genetic engineering methods have been used as strategies to improve crop drought resistance [7]. Drought-stress effects on grain yield might be minimized by application of optimum management methods related to seeds sowing time, plant genotype, soil, and nutrient management. However, the adoption of transgenic plants with drought-tolerant profiles is likely the most popular drought stress reduction strategy. As a result, many molecular and genetic methods are being used to generate novel drought-resistant plants [2]. Nonetheless, the lack of a complete molecular basis for drought perception, signal transduction, and stress adaptation presents a major challenge to genetic methods of drought tolerance [7]. Therefore, more sophisticated research is needed to decipher the complex mechanisms of drought tolerance in plants and identify important functional machinery that can be used as tools for engineering drought-resistant crops. Other strategies that can improve agricultural stress tolerance, include the application of exogenous regulators and synthetic hormones, as well as more modern methods of irrigation, breeding, planting, mulching, contouring, but all these techniques take long time to develop and are capital-intensive [8]. There is a considerable need for developing sustainable and low-environmental-impact alternative strategies to deal with the impacts of drought. For example, use of specific microorganisms can significantly improve abiotic stress tolerance and provide other added benefits that include boosting plant development, enhancing nutrients acquisition, reducing plant diseases and pathogen attacks and as a results reduce usage of agrochemicals [9]. This review article focuses on use beneficial rhizobacterial communities as the key environmentally friendly elicitors of drought tolerance in plants.

Plants are naturally surrounded by a diverse population of microorganisms in the rhizosphere. The rhizosphere is an ecological hotspot of strong dynamic interactions between plants and microbial activity, and it contains thousands of different species of bacteria, fungi, protozoa, and other microorganisms. Rhizobacteria comprise various groups of bacteria that are directly influenced by root exudates and are considered the most abundant microorganism in the rhizosphere [10]. Among the rhizobacteria are a beneficial bacterial species referred to as Plant Growth-Promoting Rhizobacteria (PGPR). PGPR can colonize plant roots, significantly increase soil fertility, promote plant growth and development, and enhance crop yield. Furthermore, these PGPR have the potential to benefit their host plants by promoting a variety of direct and indirect responses to overcome the effects of drought stress and, as a result, enable plants to survive these stressful conditions [11]. The review highlights the mechanisms used by PGPR, to induce drought stress tolerance in a sustainable environmental-friendly manner.

## Effect of plant growth promoting rhizobacteria in drought stress tolerance

### Effect of drought stress on plant growth

**Fig. 1:**
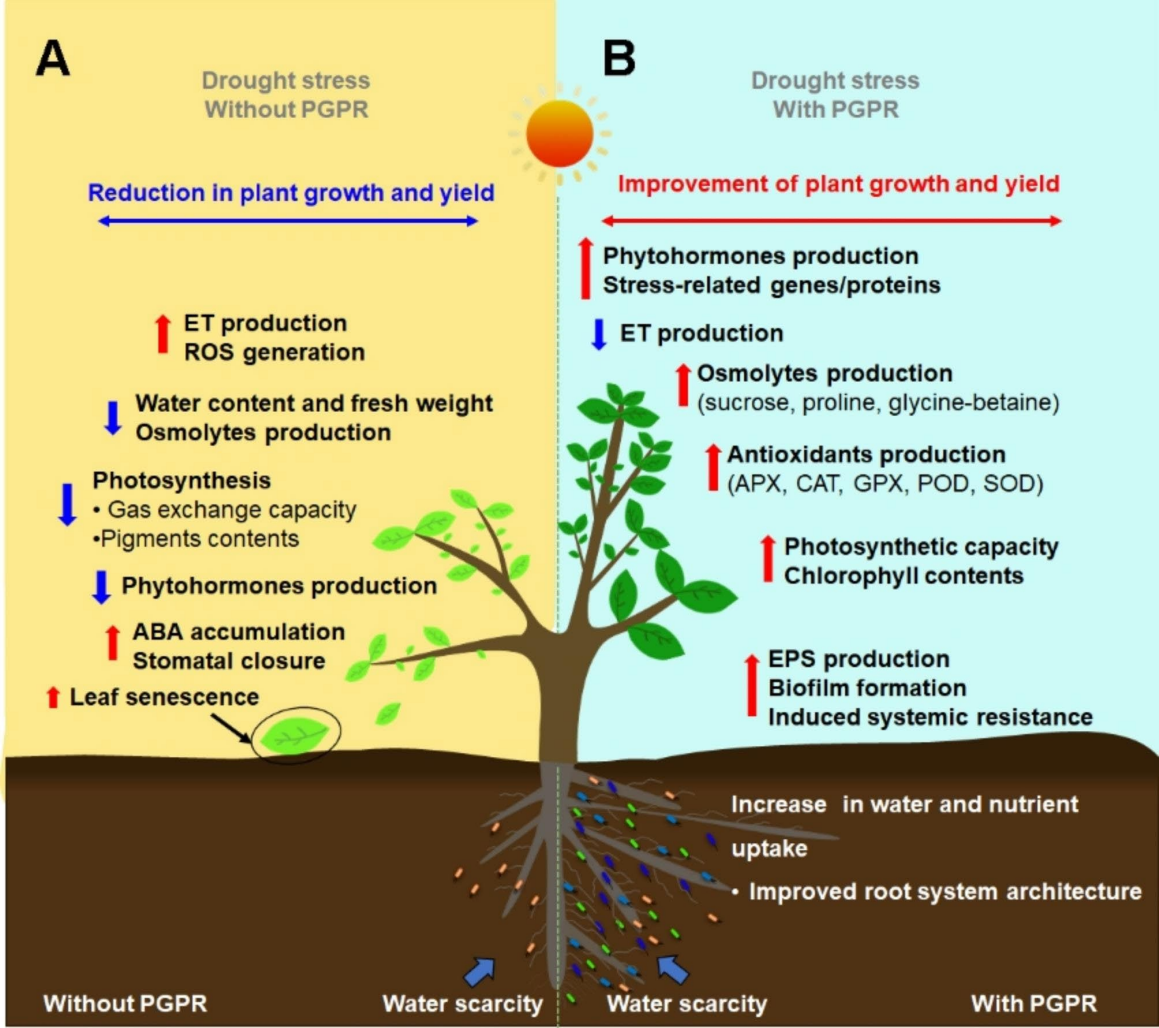
A comparison of the effects of drought stress in presence or absence of plant growth promoting rhizobacteria. **A**. Major processes adopted by plants in response to drought stress. The impacts of drought stress on plant physiological and biological processes include accumulation of ABA and stomatal closure to reduce water loss, increased phytohormone imbalance, ROS accumulation, decreased PSII functional antenna cross-section, and reduced root system development, which negatively affect water content and osmolytes production. Drought stress also increases ethylene production which increases leaf abscission and senescence. **B**. Role of PGPR in alleviating drought stress impact in host plant. PGPR positively affect vegetative parameters and physio-biochemical traits of inoculated plants via various processes including amelioration of root system development, which improve water and nutrients uptake, enhanced production of osmolytes that play a role in osmotic adjustment and enable the maintenance of cell turgor for plant survival. PGPR enhance the accumulation of antioxidants enzymes to decrease ROS content in plants. PGPR ameliorate photosynthetic activity, pigments production and phytohormones homeostasis. PGPR induce expression of stress-related genes and accumulation of proteins involved in metabolite synthesis to help plants cope with drought stress. PGPR: Plant Growth Promoting Rhizobacteria; ABA: Abscisic acid; APX: ascorbate peroxidase; CAT: catalase; EPS: Exo-polysaccharides: ET: ethylene; GPX: glutathione peroxidase; POD: peroxidase; SOD: superoxide dismutase; ROS: Reactive oxygen species

Drought stress is an abiotic stress factor that has become more intense in recent decades, and it is predicted to wreak serious havoc on more than half of the world’s agricultural land by 2050, thus affecting plant productivity and global food security. It is estimated that drought stress reduces cereal, rice, wheat and maize yields by 10%, 25%, 21%, and 40% respectively [12]. To make matters worse, a 60% increase in wheat production is needed to fulfill the rising market demand due to the growing world population [13]. Drought is described as a physiological state where plant growth and yield are hampered by low water potential and tissue turgor. The subsequent stress reduces seed germination, leaf area, leaf expansion, cell division and elongation, which decrease photosynthesis activity, resulting in reduced plant growth and yield (Fig. 1. A). Drought can cause a reduction in water content in plants, which is typically accompanied by a series of adaptation processes such as production of osmolytes like proline, sucrose, polyamines, and extracellular polysaccharide (Fig. 1. A). Evidently, drought limits the plant’s water potential and induces stomatal closure to save water in the leaves, which affects the flow of nutrient and availability to the roots (Fig. 1. A [12, 14],). Water scarcity affects leaf gas exchange owing to stomata closure, which limits tissue growth, reduces fresh and dry biomass production, transpiration rate, and consequently slows down the photosynthetic activities (Fig. 1. A [3, 15], In addition, drought stress decreases the abundance of proteins involved in photosynthesis such as ribulose-1,5-bisphosphate carboxylase (Rubisco) activase and Rubisco large subunit-binding protein, which play a role in making the active site of Rubisco catalytically competent and assembling Rubisco in chloroplasts respectively [16]. The decreased abundance of these proteins indirectly results in degradation of Rubisco and consequently in diminished CO_2_ fixation [16, 17]. Photosynthetic pigments, chlorophyll a and b are reduced under drought, which lowers the levels of total chlorophyll and chlorophyll-binding proteins [18]. Drought stress disrupts the major components of the photosynthetic apparatus (Fig. 1. A), including the photosynthetic pigment-protein complexes, chloroplast shape, structural organization of thylakoids and electron transport chain, respiration, translocation, ion uptake, sugar and nutrient metabolism, and activity of Rubisco, which reduces carboxylation capacity regulated by stomata [19]. This can increase carbohydrate accumulation and initiate the generation of reactive oxygen species (ROS) that are toxic to plant organelles, resulting in oxidative stress in membrane lipids, denaturation of proteins, inhibition of enzyme activities, and disturbance in water uptake [20].

For example, maize seedlings grown under drought stress reduced the net photosynthesis, transpiration rate, stomatal conductance, and water use efficiency by about 33%, 38%, 26% and 51% respectively [21]. However, plant sensitivity to drought is dependent on plant species and the developmental status as well as the duration and degree of the stress. In legumes, drought stress decreases transpiration rate, resulting in reduction of the rate of xylem translocation and enzymatic activity, and limiting the symbiotic nitrogen fixation rate [22]. Drought stress decreases grain growth and yield by limiting the number of tillers, spikes, and grains per plant in barley (*Hordeum vulgare*). Therefore, water scarcity affects plant nutrition acquisition, and can cause nutritional deficiencies, which in return can affect the physiological activities in plants [23]. Drought-related features such as root properties and ABA concentration have been employed as markers of drought resistance [15]. In drought conditions, roots send stress signals to the shoots through variations in abscisic acid (ABA), cytokinin, and other substances involved in the root-shoot signaling pathway, leading to induction of physiological changes to adapt to or face the stress effect (Fig. 1. A;). Because of the disruption of the cell membrane or cell turgor, drought stressed plants accumulate ABA levels in their tissues to regulate stomatal closure. For instance, *Vitis vinifera* modulates stomatal closure and then accumulates ABA to keep the stomata closed in response to water shortages [24]. Drought tolerance is mediated by the transcription factor dehydration-responsive element-binding protein 1 A (DREB1A), which interacts with the dehydration-responsive element (DRE) [25]. Drought stress activates the ABA-dependent signaling pathway and enhances ABA production, which stimulates the 9-cis-epoxycarotenoid dioxygenase (NCED) gene expression in *Arabidopsis.* ABA acts as a stress hormone to help plants cope with drought stress [26].

### Mechanisms used by PGPR to alleviate drought stress

Plants undergo a variety of sophisticated physiological, morphological, cellular, biochemical, and molecular processes to respond and adapt to drought stress [3, 13]. Extensive research is being conducted to develop strategies that help plants to cope with drought stress. However, most of these technologies are costly and time consuming [14]. In recent years there has been increasing interest in agricultural production methods that are environmentally friendly. PGPR inoculants are a sustainable alternative strategy for not only alleviating the drought stress impacts, but also promoting plant growth and development [27]. Although the exact mechanisms of PGPR in mediating drought stress tolerance in plants are largely unknown, there are several logical explanations that can lead to the observed outcomes (Fig. 1B). Many studies have demonstrated that under drought conditions, PGPR can act as biofertilizers that directly promote plant growth by improving acquisition of certain nutrient through different processes such as nitrogen fixation, mineral solubilization, phosphate and potassium absorption, siderophore production and iron sequestration ((Fig. 1B; Table 1) [28]. For example, inoculation of wheat seedlings with *Azospirillum brasilense Sp245* strain under drought stress significantly increased potassium (K), magnesium (Mg), and calcium (Ca) contents in the grains, and improved relative water content and water potential, which enhanced mineral quality and grain yield [29]. In addition, PGPR can improve drought tolerance capacities by stimulating the production of drought-tolerant substances, such as, amino acids, 1-aminocyclopropane-1- carboxylate (ACC) deaminase [28], volatile organic compounds (VOCs;), sugars (that prevent degenerative processes), bacterial exopolysaccharides (EPS), and phytohormones like auxin or indole-3-acetic acid (IAA), cytokinin (CK), abscisic acid (ABA), ethylene (ET) salicylic acid (SA) (Fig. 1. B; 14,30,31]). These stress-fighting agents can prevent the accumulation of reactive oxygen species (ROS) by production of antioxidant enzymes [12]. For instance, Kasim et al., [13] demonstrated that inoculation of wheat seedlings with *Bacillus amyloliquefaciens* strain *5113* and *Azospirillum brasilense* strain *NO40* increased IAA and EPS production, and enhanced antioxidants activities, which improved wheat biomass and growth. Other study showed that *Bacillus pumilus* enhanced root length, accumulation of metabolites and antioxidants enzymes, while decreasing production of ROS molecules, such as hydrogen peroxide and superoxide anion radicals.

**Table 1:** Contributions of plant growth-promoting rhizobacteria to alleviation of drought stress in plants

PGPR strains	Drought stressed plant species	Effects of PGPR in drought stressed plants	References
Rhizobium	*Phaseolus vulgaris* L.	Improved nutrient content and plant yield	[32]
*Paenibacillus polymyxa*, *Rhizobium tropici*	*Phaseolus vulgaris* L.	Increased nodule number, shoot dry weight, and plant height. Increased abscisic acid (ABA) levels in the leaves and reduced cytokinin (CK) levels and stomata closing	
*Azospirillum brasilense* strain *Cd*	*Phaseolus vulgaris* L.	Increased root length, root projection and root fresh weight	[33]
*Rhizobium elti* (Overexpressed trehalose-6-phosphate synthase gene)	*Phaseolus vulgaris* L.	Enhanced nodules, nitrogenase activity, biomass, and grain yield	[34]
*Bradyrhizobium japonicum*	*Glycine max* L.	Enhanced nodulation, production of indole-3-acetic acid (IAA) and exopolysaccharides (EPS)	
*Sphingomonas sp*., strain LK11	*Glycine max* L.	Increased ABA and jasmonic acid (JA) contents, plant biomass, photosynthetic pigments, production of glutamine, glycine, and proline	[35]
*Pseudomonas putida* strain H-2-3	*Glycine max* L.	Modulated hormonal and antioxidants regulation, and improved crop productivity	
*Variovorax paradoxus* strain 5 C-2 (containing the enzyme 1-aminocyclopropane-1- carboxylate (ACC) Deaminase)	*Pisum sativum* L.	Reduced ethylene (ET) production, increased nodulation, seed nitrogen content, xylem ABA concentration, water content and pea yield	[36]
*Pseudomonas fluorescens* biotype G (ACC-5; containing ACC Deaminase)	*Pisum sativum* L.	Induced longer roots and increased water uptake capacity	[37]
Pseudomonas *entomophila* strain BV-P13, *P. stutzeri* strain GRFHAP-P14, *P. putida* strain GAP-P45, *P. syringae* strain GRFHYTP52, *P. monteilli* strain WAPP53	*Zea mays L.*	Improved plant biomass, relative water content, root adhering soil/root tissue ratio, relative water content and leaf water potential, increased proline, sugars, and free amino acids production	[38]
*Pseudomonas putida* strain FBKV2	*Zea mays* L.	Enhanced root and shoot growth and dry biomass weight, and reduced stomatal conductance	[39]
*Azospirillum lipoferum*	*Zea mays* L.	Enhanced accumulation of free amino acids and soluble sugars	
*Bacillus subtilis*, *Bacillus amyloliquefaciens*, *Bacillus licheniformis*, *Bacillus thuringiensis*, *Paenibacillus favisporus*	*Zea mays* L.	Increased root adhering soil/root tissues ratio, relative water content, leaf water potential, plant biomass, proline, total soluble sugars, free amino acids. Reduced leaf water loss, electrolyte leakage and activity of antioxidant enzymes	[40]
*Burkholderia phytofirmans strain PsJN* and *Enterobacter sp.*, strain *FD17*	*Zea mays* L.	Enhanced root and shoot biomass, leaf area, photosynthesis, and chlorophyll content	[41]
*Azospirillum brasilense* strain BR11005	*Zea mays* L.	Increased root growth, total aerial biomass, foliar area, leaf relative water content, and proline	[42]
*Azospirillum brasilense* (Overexpressed trehalose-6-phosphate synthase gene)	*Zea mays* L.	Increased maize biomass	
Azospirillum lipoferum	*Zea mays* L.	Increased levels ABA and IAA	[43]
*Achromobacter* *piechaudii* Strain ARV8	*Lycopersicon esculentum* L. and *Capsicum annuum*	Reduced ET production and increased fresh and dry weights	[44]
*Bacillus subtilis* and *Paenibacillus Illinoinensis*	*Capsicum annuum*	Enhanced root length, transpiration, cell turgor, net photosynthetic rate, and proline content	[45]
*Bacillus licheniformis* strain *K11*	*Capsicum annuum*	Produced IAA and 1-aminocyclopropane-1- carboxylate (ACC) deaminase, increased root and shoot length and dry weight	
The PGPR *Variovorax paradoxus* 5 C-2 and Arbuscular mycorrhizal (AM) fungus *Rhizophagus irregularis*	*Lycopersicon esculentum* L.	Enhanced photosynthetic rate, osmolytes, root water conductivity, and oxidative phosphorylation status	[46]
*Azospirillum brasilense* strain *Sp245*	*Lycopersicon esculentum* L.	Induced lateral and root hair development	[47]
*Bacillus polymyxa*	*Lycopersicon esculentum L.*	Increased phosphate solubilization and proline accumulation	[48]
*Bacillus sp*	*Lactuca stiva* L.	Improved photosynthesis apparatus	
*Pseudomonas mendonica*	*Lactuca stiva* L.	Increased phosphatase activity in roots and proline accumulation in leaves	[49]
*Glucoacenatobacter diazotrophicus*	*Saccharum officinarum*	Induced drought stress responsive genes, and biosynthesis of ABA and ET	[50]
*Azospirillum* isolates, Azo 195, Azo 249, Azo 274	*Saccharum officinarum*	Increased IAA production, root dry mass and shoot height	[51]
*Pseudomonas putida* strain MTCC5279 (RA)	*Cicer arietinum L.*	Down-regulated stress response gene, increased water content, osmolyte accumulation, and germination rate	
*M. mediterraneum* strain LILM10	*Cicer arietinum* L.	Increased nodule number, shoot dry weight and grain yield	[52]
*Enterobacter sp.*, *Bacillus sp.*, *Bacillus thuringiensis*, and *Bacillus megaterium*	*Thymus vulgaris*, *Lavandula dentate*, *Salvia officinalis*, and *Santolina chamaecyparissus*	Enhanced nutrient uptake, stomatal conductance, ACC deaminase, and proline	[53]
*Enterobacter sp*., *Bacillus sp*., *Bacillus megaterium*, *Bacillus thuringiensis*	*Lavandula dentata* L.	Improved total nitrogen (N) and phosphate content in soil, foliar nutrient content, and dry root and shoot biomass	[54]
*Bacillus thuringiensis*	*Lavandula dentate*	Enhanced IAA and proline, and decreased antioxidant enzymes	[55]
*Azospirillum sp.*	*Triticum Aestivum L.*	Enhanced IAA production, root growth, lateral roots formation, and increased uptake of water and nutrients	[56]
*Burkholderia phytofirmans* strain PsJN	*Triticum Aestivum L.*	Improved water use efficiency, grain yield, ionic balance, photosynthetic rate, chlorophyll content, and antioxidant level	[57]
*Pantoea alhagi*	*Triticum Aestivum L.*	Increased soluble sugars, IAA, siderophore, EPS, ammonia, and protease production, and decreased chlorophyll degradation	[58]
Consortia containing: *Pseudomonas jessenii* strain R62, *pseudomonas synxantha* strain R81 and *Arthrobacter nitroguajacolicus* strains YB3 and YB5	*Oryza sativa* L.	Increased proline accumulation	[59]
*Trichoderma harzianum*	*Oryza sativa* L.	Increased stomatal conductance, net photosynthesis, and concentration of stress induced metabolites	[60]
*Azospirillum Brasilense* interacted with arbuscular mycorrhizal fungi (AMF)	*Oryza sativa* L.	Increased shoots fresh weight, plant vigor, stomata conductance, and photosynthetic status	[61]
*Bacillus subtilis*	Platycladus orientalis	Increased ABA levels in shoots and stomatal conductance	
*Sinorhizobium medicae*	*Medicago truncatula*	Enhanced potassium and N contents, root nodulation, osmolytes production, translational regulation, and delayed leaf senescence	[62]
*Pseudomonas putida* strain MTCC5279 (RA)	*Cicer arietinum* L.	Increased seed germination, root and shoot length, lateral roots, fresh and dry weights, and reduced ET biosynthesis	
*Azospirillum brasilense* and *Pantoea dispersa*	*Cistus albidus* L.	Increased dry root and shoot weight	[63]
*Bacillus subtillis* strain B26	*Brachypodium distachyon Bd21*	Enhanced root and shoot biomass, seed yield, total soluble sugars, and starch contents. Upregulated drought responsive genes	
*Pseudomonas putida* Strain GAP-P45	*Helianthus annuus*	EPS production and increased total dry biomass	[64]
*Bacillus thuringiensis* interacted with the fungus *Glomus intraradices*	*Retama sphaerocarpa*	Enhanced root development and water transport	[65]
*Pseudomonas fluorescens*	*Catharanthasus roseus*	Improved plant growth	[66]
*Variovorax paradoxus* Strain 5 C-2	Ornamental species	Reduced ET production	[67]
*Pseudomonas putida* and *Bacillus megaterium*	*Trifolium repens*	Increased IAA production, root and shoot biomass and water content	[67]
*Rhizophagus intraradices*, *Bacillus megaterium*, and *Pseudomonas putida*	*Trifolium repens*	Increased plant nutrient uptake and relative water content, and reduced stomatal conductance, electrolyte leakage, and antioxidative activities.	[69]
*Pseudomonas fluorescens*	*Catharanthus roseus*	Increased IAA production, fresh and dry weights	[66]
*Micrococcaceae* strain HW-2	*Arundo donax*	ACC deaminase and siderophore production, enhanced water retention capacity, root and shoot growth and plant biomass	
*Azospirillum brasilense* and *Pantoea dispersa*	*Pinus halepensis*	Enhanced total carbohydrates, microbial biomass carbon, and soil nutrient uptake, water potential and seedling growth, and antioxidant enzyme activities	[70]

#### PGPR induced root and shoot growth

The intimate interactions between plants and PGPR required for better acquisition of nutrients and water absorption are complex. PGPR have been shown to boost root length and density, which facilitates nutrient and water uptake, promotes crop yield, and maintains the osmotic balance in the host-plant through the relative water content [14]. Roots produce exudates that contain a variety of high and low-molecular-weight compounds including a large amount of carbohydrates, polysaccharides, lipids, and amino acids that provide nutrients for microbes, resulting in increased microbial populations around the roots. Root exudates may also function as signaling molecules for the microbes associated with the roots, and as a result actively attract microbes to the roots [8]. For instance, it has been reported that malic acid exudate can attract the beneficial rhizobacteria *Bacillus subtilis* to the root [71]. PGPR colonize plant roots, which leads to direct or indirect plant growth promotion, and protection from drought stress and disease. Several PGPR influence plant development through interactions with plants and their metabolic activities. For example, Farooqi et al., [26] demonstrated that during vegetative development, drought stress can reduce around 41% of shoot growth in the absence of PGPR. However, after PGPR inoculation, drought stress reduced the plant shoot growth by only 18%. The improved drought tolerance may be due to changes in the root zone organization resulting from plant- PGPR interaction. The root system architecture is an adaptive feature to drought conditions. Plants adapt their root development to cope with drought stress by increasing the root: shoot biomass ratio to assist them in absorbing more water [3].Thus, under drought conditions, a larger and deeper root system can increase plant yield. In fact, stressed plants use the root to increase the surface area in contact with soil moisture, thereby boosting the hydraulic conductivity. As a result, the root system enhances water and nutrient acquisition, allowing plants to withstand drought stress. Root colonizing PGPR may be used as bio-fertilizers to increase root length, which results in a more effective root system with a larger root surface area, a higher rate of lateral root elongation, and a considerable number of lateral roots. This can boost plant resistance to drought stress factors and enhance overall plant biomass output.

A wide range of common PGPR species that colonize plant roots and enhance the plants’ ability to cope with drought stress include *Pseudomonas, Bacillus, Rhizobium, Enterobacter, Azospirillum, Azotobacter, Arthrobacter, Mesorhizobium, Xanthomonas, Acinetobacter, Alcaligenes, Burkholderia, Erwinia*, and *Flavobacterium* [72]. Naveed et al. [11] showed that maize inoculated with *Burkholderia phytofirmans* had increased root and shoot biomass growth under drought stress compared to non-inoculated plants. For instance, PGPR inoculated pepper plants increased the root system size by about 40%, which enhanced the water absorption and resistance to water stress. According to Tiepo et al. [30], inoculation of *Trema micrantha* plants with PGPR, such as *Azospirillum brasilense*, *Bacillus sp*., *Azomonas sp*., and *Azorhizophillus sp*., activated numerous metabolic pathways, which improved plant tolerance to drought stress. Inoculated *T. micrantha* showed enhanced root and shoot dry mass, protein, starch, photosynthesis, and carboxylation contents. Colonization of seeds with *Pseudomonads* and *Acinetobacter* induced root formation, increased nutrient uptake which was attributed to regulation of phytohormone synthesis [73]. *Bacillus megaterium* and *Pseudomonas putida*, have developed several processes to assist plants cope with drought stress e.g. they boost IAA synthesis; generate CK or reduce ET production; enhance nutrient uptake, increase shoot and root biomass, and water content [68].

#### Role of PGPR induced hormones in drought stress tolerance

During the different phases of plant growth, signaling molecules from plants and beneficial microorganisms play an important role in establishing the communication between the partners. PGPR can improve agronomic yields by inducing and/or producing plant growth regulators [31]. Plant growth regulators are commonly referred to as plant hormones, and are defined as organic compounds synthesized exogenously to govern the physiological processes of plant growth and development [31]. They were initially known for their role as plant growth regulators which promote plant growth, but later they became known for their ability to help plants to cope with environmental stresses. Synthetic or natural plant growth regulators can be used to improve drought tolerance in plants through their stimulatory or inhibitory effects. Several phytohormones, such as indole-3-acetic acid (IAA) or auxin, cytokinin (CK), abscisic acid (ABA), jasmonic acid (JA), salicylic acid (SA), and ethylene (ET), are well-known for their direct induction of plant development, but they also play a role in drought stress tolerance [72]. Interestingly, inoculation of plants with PGPR can also modulate plant hormone synthesis to promote plant tolerance to drought stress. PGPR produce several external plant growth regulators such as auxin or IAA [74], CK [75], ET [76], ABA [77], SA [78], and JA [79]. The phytohormonal pathways can engage in a network of interconnected responses to help plants cope with drought stress.

##### Indole-3-Acetic acid

Auxin is recognized as one of the most important plant growth regulators. Indole-3-acetic acid (IAA) is the main active natural form of auxin involved directly in plant development [80]. IAA is involved in the regulation of different biological processes connected to plant growth, including seed germination; cell division, elongation, and differentiation; gene expression; photosynthesis initiation and proliferation of lateral and adventitious roots. Among the most well-known IAA-producing rhizobacteria genera are *Pseudomonas*, *Rhizobium*, *Azotobacter*, *Arthrobacter*, *Azospirillum*, *Stenotrophomonas*, *Microbacterium*, *Sphingomonas*, and *Mycobacterium* strains [81]. Bacterial phytohormones can interfere with endogenous host-plant IAA and hence regulate plant development [82]. Beneficial bacteria can assist plants in overcoming drought stress by supplying auxin as a key plant growth regulator. About 80% of rhizobacteria can regulate IAA production in plants. Plant roots excrete L-tryptophan, a major precursor for IAA [83]. PGPR in the rhizosphere convert tryptophan to IAA which is taken up by the plants [82]. PGPR can also produce their own IAA, which is assimilated by plant cells, activates the auxin signal transduction pathway in plants, and stimulates plant cell proliferation [82]. Numerous PGPR and plants can synthesize IAA mostly via five distinct pathways [83]:

(1) IAA biosynthesis through indole-3-acetamide (IAM) is found in beneficial bacteria such as *Pseudomonas putida*, and *Pseudomonas fluorescens* [82].

(2) Tryptophan conversion to indole-3-acetonitrile (IAN) has been observed in beneficial bacteria genera such as *Ensifer* and *Rhizobium* [83].

(3) IAA biosynthesis via indole-3-pyruvic acid (IPA). This IAA biosynthesis process is found in various bacterial species like *Pseudomonas*, *Rhizobium*, *Bradyrhizobium*, *Enterobacter, Agrobacterium*, *Azospirillum*, and *Klebsiella* [84].

(4) The tryptamine pathway, in which tryptophan is first converted to tryptamine before being converted to IAM is suspected to occur in *Azospirillum* [83].

(5) The tryptophan-independent pathway is found in plants, cyanobacteria and *Azospirilla*.

The concentration of IAA fluctuates depending on the presence of different strains [82]. It has been shown that the IPA pathway is associated with epiphytic and rhizospheric fitness, while the IAM pathway is associated with phytopathogenicity [83]. However, this may not hold true for all bacteria since it has been demonstrated in pathogenic bacteria, but the IAM pathways are also found in beneficial bacteria. Bacterial IAA induce different responses in plants depending on the physiological concentration. For example, modest levels of IAA produced by PGPR can boost primary root elongation and plant growth, whereas high levels of exogenous IAA can inhibit primary root elongation and enhance lateral and root hair development [85]. IAA behaves as a reciprocal signaling molecule that modulates the PGPR’s gene expression, which is essential for rhizobacteria-plant interactions [83]. Inactivation of the gene encoding for indole-3-pyruvate decarboxylase (ipdC) in *Azospirillum brasilense* strain reduces IAA synthesis. This enzyme catalyzes the rate limiting step involving the decarboxylation of indole-3-pyruvate to indole-3-acetaldehyde which is then oxidized to IAA. Inoculation of wheat with this ipdC mutant dramatically altered development of lateral roots [86]. However, the synthesis of large amounts of IAA by PGPR was found to impede rather than enhance root development.

Drought stress frequently inhibits the synthesis of endogenous auxins, however, IAA- producing PGPR act in tandem with endogenous plant IAA to increase growth of lateral and hair roots, which increases surface area and, subsequently, boosts water and nutrient uptake and assist plants to cope with drought stress [7, 85]. This can promote auxin homeostasis and increase plant tolerance to drought stress by stimulating endogenous auxin levels and interfering with plant IAA transport. Cardoso et al. [87] showed that IAA-producing bacteria colonizing the roots of legumes improved drought tolerance compared to the un-inoculated plants. *Azospirillum brasilense* producing IAA enhanced plant tolerance to drought stress and inoculation of tomato plants with *A. brasilense* producing nitric oxide, a diffusible gas that act as a signaling molecule in IAA production, increased adventitious root development and enhanced plant’s tolerance to drought stress [47]. Similarly, under drought stress, inoculation of common bean (*Phaseolus vulgaris L*.) with *A. brasilense Cd* increased auxin production, enhanced root area and length compared to non-inoculated plants [34]. Inoculation of wheat seedlings with *Azospirillum lipoferum* strains reduced leaf water potential and increased leaf water content during drought stress, enhanced production of IAA, and improved root development and lateral root formation for water and nutrient uptake [56]. Interestingly, PGPR can regulate the plant auxin pathway through production of other regulators such as nitric oxide (NO), 2,4-diacetylphloroglucinol (DAPG), and some phytohormones like cytokinin [85]. Furthermore, many studies have suggested that bacteria that protect plants against drought stress may generate both IAA and ACC-deaminase, which act synergistically to promote plant development [88].

##### Cytokinin

Cytokinin (CK) is a key plant hormone that regulates general physiological and developmental processes such as cell division and enlargement, tissue expansion, nutrient allocation, leaf senescence, root nodule development, and auxin action [74]. CK plays a role in mediating signaling from roots to shoots. In addition, CK is a double-edged phytohormone that negatively regulates many aspects of plant growth, for example it can inhibit lateral root formation and primary root elongation [89]. The effect of CK on plant growth is typically regulated by the CK concentration. A high concentration of CK can prevent root development, whereas low levels of cytokinin can cause the plant to perish, in fact CK-deficient plants develop smaller apical meristems [90]. The production and function of CK in plants is well documented, for example, in *Arabidopsis thaliana* the CK signal is received by three identical histidine kinases receptors, AHK2, AHK3, and AHK4 [91]. However, despite the bacterial genes implicated in CK biosynthesis pathways having been identified *in silico*, their roles and functions have yet to be determined.

CK plays an important role in the drought tolerance of higher plants [74]. During drought stress, endogenous levels of CK usually decline, which can boost ABA levels and enhance shoot responses to ABA in stressed plants. These stress-induced changes in ABA and CK levels promote early leaf senescence and abscission, and reduction of water loss through transpiration under drought stress [92]. CK plays a key role in water loss reduction by increasing stomatal resistance and water uptake by a developing large and deep root system, increasing osmo-protectant synthesis and osmolytes accumulation. However, in certain cases of drought stress, increased CK concentration in the xylem sap may delay the reduction of stomatal aperture or directly stimulate stomatal opening and lower stomatal sensitivity to ABA [93]. In addition, Nishiyama et al., [94] showed that reduced endogenous CK levels do not always imply increased sensitivity to ABA. CK-deficient mutant *Arabidopsis* plants maintained their ability to regulate the ABA:CK ratio and stomatal opening for carbon dioxide absorption indicating that other mechanisms besides CK are involved in regulation of stomatal response under water deficit. For example, during drought stress, CK and ABA might cooperate to collectively exert antagonism on auxin, thus stimulating the development of the primary root for seeking a water source [95]. Therefore, cross-talk between different hormones influences growth and development of the plants. This hormonal balance is influenced by the concentrations of several growth regulators as well as by environmental cues.

PGPR can aid growth by modifying this hormonal balance in the host-plant, which can play a role in the host-plant response to drought stress. PGPR generated CK stimulate plant cell division, cell expansion, root and shoot growth, root hair proliferation, and increase root surface area [91]. The ability of PGPR to synthesize CK as a plant growth regulator is one of the proposed mechanisms by which PGPR increase plant development and help plants cope with drought stress. However, little information is available on the potential role of cytokinin-producing PGPR on plant growth under drought stress conditions [96]. Given that the declining concentrations of CK during drought are associated with plant adaptations to drought stress through promotion of root: shoot biomass ratio and enhanced sensitivity to ABA, the mechanism through which CK producing PGPR can promote drought stress is not clear. This is supported by the findings of Arkhipova et al., [97], who observed that inoculation of lettuce seedlings with a CK-producing *Bacillus* (strain IB-22) lowered root: shoot biomass by promoting shoot growth and shortening roots. Therefore, these lettuce seedlings did not develop the expected drought stress adaptations, but the shoot biomass increased under drought stress. In this study, the effects CK on stomatal conductance were masked by increased ABA production by the *Bacillus* species (strain IB-22), which shows the complexity of disentangling PGPR functions and ascribing single outcomes to their application.

Several PGPR strains known for their ability to produce CK include *Pseudomonas*, *Bacillus*, *Bradyrhizobium*, *Azospirillum*, and *Arthrobacter* species. *Bacillus subtilis* producing CK increased the endogenous content of CK and promoted lettuce development under drought conditions [98]. CK-producing *Bacillus subtilis* had a favorable effect on *Platycladus orientalis* (oriental thuja) plant development under drought stress [75]. In this study, drought stress alone reduced shoot CK levels by 39.14%, but inoculation with cytokinin-producing *Bacillus subtilis* reduced the inoculated stressed plants’ CK by only 10.22%. When compared to non-inoculated plants, inoculated seedlings exhibited increased relative water content, improved leaf water potential, increased root dry weight and shoot dry weight by 13.99% and 19.23% respectively, and enhanced production of root exudates such as sugars, amino acids, and organic acids. Thus, CK-producing PGPR can alleviate the drought stress in *Platycladus orientalis* seedlings and promote their growth. However, the CK-producing *B. subtilis* used several mechanisms to promote plant growth and enhance drought tolerance under water deficit.

##### 1-Aminocyclopropane-1-carboxylate deaminase and ethylene

Ethylene (ET), an essential gaseous plant growth hormone produced endogenously by plants, is required for normal plant development even at extremely low concentration levels [99]. ET controls a variety of developmental processes in plants, including shoot and root differentiation, lateral root formation, seed germination, flowering induction, senescence, leaf abscission, and fruit maturation. However, ET can also prevent root growth, nodulation, and auxin transport [100].

PGPR synthesize IAA from tryptophan excreted by plant roots. IAA molecules can induce transcription of the gene encoding 1-aminocyclopropane-1-carboxylate synthase (ACS) enzyme, resulting in enhanced concentrations of 1-aminocyclopropane-1-carboxylate (ACC), the immediate precursor of ET, and hence increased the production of ET in plants and promotion plant cell development [101]. Thus, the regulation of ACC biosynthesis pathway genes regulates ET production in plants. In addition, higher plants and some PGPR use the S-adenosylmethionine (S-AdoMet), generated from methionine, and converted it to ACC by the action of ACC-synthase and ACC-oxidase to synthesize modest amounts of ET [99]. This small quantity of ET plays a key role in plant protection by increasing stress-related genes activities [101]. However, some of the ACC released into the rhizosphere is reabsorbed by the roots and converted into ET. This accumulation of ET inhibits root growth, which reduces the bioavailability of water and nutrients, which leads to more stress. Therefore, in addition to being a plant growth regulator, ET also acts as a growth inhibitor or a stress hormone under adverse conditions such as salinity, heavy metal toxicity, and drought stress [102]. Under stress conditions, the endogenous amounts of ET are significantly increased which may reach levels that adversely limit plant development [102]. Plants enhance the synthesis of endogenous ET in response to stress stimuli, resulting in reduced root and shoot development, and plant growth retardation [102]. ET may modulate leaf function throughout the leaf life cycle and mediate drought-induced senescence. For example, high levels of ET cause cell defoliation, which inhibits root and stem growth and results in early senescence, subsequently leading to yield reduction [102]. Also, under stress conditions, high levels of ET in legumes can cause premature senescence and arrest some physiological processes such as root growth and nitrogen fixation.

Some PGPR strains have been shown to produce the enzyme ACC deaminase. This enzyme sequesters and catalyzes the conversion of ACC to ammonia and α-ketobutyrate instead of being converting it to ET [103]. These two products can be used as carbon and nitrogen sources for the host plant [76]. This cleavage decreases ACC and ET levels in the rhizosphere, which lead to a reduction in endogenous ET levels in plants and subsequently, minimize the inhibitory effect of higher ET concentrations [104]. Thus, ACC deaminase producing PGPR improve multiple physiological and biochemical features in plants, including root and shoot growth, mineral nutrients uptake, membrane stability, photosynthetic pigment production, rhizobial nodule formation, and mycorrhizal colonization in various crops [82]. Therefore, PGPR that produce ACC deaminase are involved in the direct negative regulation of endogenous ET levels in plants and in stimulating plant growth [76]. The ability ACC deaminase producing PGPR to decrease the amount of ACC available for ET synthesis, can reduce the harmful effects caused by the high concentration of ET and aid in reestablishing the root growth, promoting plant development and tolerance to drought stress [82]. Numerous studies have shown that in the absence of bacterial ACC deaminase, ET levels in plants rise, which inhibits cell proliferation by restricting transcription of auxin response factors and IAA production [82]. However, in the presence of ACC deaminase less ET is generated, transcription of auxin response factors is not blocked, and the produced IAA can boost cell proliferation without simultaneously enhancing ET production. As a result, ACC deaminase reduces growth inhibition by ET while also allowing IAA to promote plant growth, both in the presence and absence of plant stress [82]. Therefore, inoculating plants with ACC deaminase producing PGPR strains can be beneficial in reducing the detrimental impact of stress-induced ET on root growth. Most of the studies involve PGPR root inoculation and colonization, however, spraying drought sensitive rice plants with IAA producing *Bacillus megaterium* PB50 was thought to have induced ACC deaminase production which in turn reduced ET levels in the plant resulting in drought stress tolerance in rice plants [105].

Several studies in which PGPR inoculated plants were exposed to environmental stress showed that the ACC deaminase gene was upregulated. Therefore, another strategy to alleviate drought stress through ACC deaminase is to generate transgenic plants that contain the gene encoding for ACC deaminase enzyme [101]. For example, transgenic tomato plants expressing a bacterial ACC deaminase gene from *Enterobacter cloacae* UW4 or *P. putida* UW4, showed increased plant tolerance to various stress factors and decreased level of stress ET [106]. Several bacterial species that express the *acdS* gene, which encodes ACC deaminase enzyme, play other significant roles in plant growth, such as, higher expression of stress-related genes *Cadhn*, *VA*, *CaPR-10* and *sHSP*, and mitigation of drought stress in pepper plants [107]; improved the root system, increased lateral root number, enhanced water and nutrient uptake, stimulated root–shoot length and biomass, resulting in better growth and yield production in drought-stressed wheat; enhanced the fresh and dry weights of drought stressed tomato and pepper seedlings and reduced ET production [44]; increased seed number, yield, seed nitrogen accumulation, biomass production, photosynthetic rate, and decreased stomatal resistance, improved water-use efficiency, as well as restored nodulation rate under drought stress [108]. The rhizobacteria strains exhibiting ACC deaminase production to enhance drought tolerance, belong to a wide range of genera such as *Pseudomonas, Bacillus, Enterobacter, Azospirillum, Rhizobium, Acinetobacter, Agrobacterium, Achromobacter, Alcaligenes, Burkholderia, Ralstonia*, and *Serratia* [108].

##### Abscisic acid

Abscisic acid (ABA) is a key plant growth regulator that is involved in plant development, plant defense and abiotic stress responses. ABA plays an important role in various biological processes that are critical to growth and development in plants such as seed germination and maturation, reproduction, and senescence. ABA activates drought-responsive signaling pathways and modulates physiological and biochemical adaptive drought stress responses in plants [109]. Under normal conditions, ABA level is low, and the activity of ABA receptors, such as sucrose non fermenting (Snf) 1-related protein kinase 2s (SnRK2), are inhibited by the action of 2 C protein phosphatases (PP2C) [110]. However, under drought stress, ABA level increases and then it binds to the ABA receptors, which in turn induces structural changes in the ABA-receptor complex that allow them to sequester PP2Cs [111]. Therefore, activated ABA receptors phosphorylate downstream targets and trigger ABA to act as a growth inhibitor, triggering a feedback mechanism that regulates plant stress response, stomatal responsiveness, proline synthesis, gene expression, and several adaptive physiological changes [112].

ABA synthesis is initiated following the perception of stress signals by the plasma membrane and ABA is predominantly produced in vascular tissues and acts in distant guard cell responses [113]. All plants respond to drought stress by accumulating ABA, which plays an important role in regulating stress adaptation mechanisms. ABA is a stress hormone that regulates gene expression and serves as a signal for the initiation of several processes, that lead drought stress tolerance [3]. Its involvement in gene activation and signaling can trigger the synthesis of many other phytohormones, such as IAA, ET, and CK [114]. These phytohormones improve hormonal regulatory patterns in plants, photosynthetic rate, plant growth and development under drought stress. When subjected to drought, plants regulate ABA biosynthesis by activating several ABA-responsive genes. ABA induces most drought inducible genes that play a role in protecting plant cells. Both ABA-dependent and ABA-independent transduction cascades and regulatory mechanisms govern the drought stress response, participate in drought stress signaling and in expression of water stress-induced genes [115]. ABA-dependent signaling pathways are important for stress-responsive gene expression under drought, salt, and osmotic stress. Furthermore, ABA also maintains the hydraulic conductivity of plant roots and shoots, allowing them to better uptake soil moisture and sustain cell turgor potential. This can cause an increase in antioxidant activities and accumulation of compatible osmolytes, which improves drought tolerance [116]. ABA controls the occurrence of complicated events involved in the induction of stomatal closure, which is an important water-conservation response [110]. This phenomenon can decrease the inflow of CO_2_ into the leaves, and spare more electrons for the formation of reactive oxygen species, which reduce the transpiration rate [3]. Typically, through its effect on stomatal closure and control of transpiration rate, ABA is involved in the processes conferring drought tolerance in plants. Furthermore, ABA can interact with JA and nitric oxide to boost its potential for decreasing stomatal conductance. In addition, an increase in ABA and a decrease in CK levels under water deficit, enhances stomatal closure and minimizes water loss through transpiration [92]. Under drought stress, ABA affects different aspects of plant growth, such as inhibiting leaf surface area expansion, reducing photosynthesis, decreasing lateral root initiation, enhancing the primary root length and density, and decreasing the shoot-to-root ratio to improve the uptake of water and nutrient from soil [117]. There is a negative relationship between the capacity of dehydrated rice leaves to accumulate ABA and leaf biomass. For example, increased levels of ABA are linked to decreased grain set in maize and kernel development in wheat, as well as a slower rate of endosperm cell division, when the plants are grown in water deficit conditions [118].

Several PGPR species are known to induce drought stress tolerance through the ABA pathway, for example *Azospirillum brasilense Sp245* and endophytic *Azospirillum lipoferum* enhanced ABA levels, which alleviated drought stress effects in the inoculated *Arabidopsis* and maize plants [43]. *Phyllobacterium brassicacearum STM196* beneficial strain increased ABA content in inoculated *Arabidopsis* plants, which reduced leaf transpiration and improved osmotic stress tolerance in the plants [119]. Inoculation of *Platycladus orientalis* seedlings with cytokinin-producing *Bacillus subtilis* strain resulted in higher ABA levels in shoots and increased stomatal conductance, conferring resistance to drought stress [75]. Treating mustard seedling with a *Bacillus marisflavi CRDT-EB-1* strain that produced ABA precursors, xanthoxin and xanthoxic acid, induced stomata closure and caused the plants to be drought tolerant [120]. Wheat plants inoculated with *Dietzia natronolimnaea* showed increased expression of genes involved in the ABA-signaling cascade and various antioxidants enzymes, suggesting that ABA signaling is involved in this PGPR’s plant stress tolerance mechanisms. ABA is a critical plant regulator hormone that maintains plant tissue homeostasis under diverse abiotic stresses by controlling the biosynthesis of a large variety of proteomes in the plant.

##### Salicylic acid

Salicylic acid (SA) is a plant hormone that is largely known for its role in systemic acquired resistance (SAR) and induced systemic resistance (ISR) in which plants treated with microorganisms show resistance to several disease causing plant pathogens [121]. In addition, SA has been shown to promote plant growth by regulating nitrogen and proline metabolism, flowering induction, stomata opening and closing, photosynthesis and respiration, synthesis of glycine-betaine (GB), antioxidant defense enzymes, and ET [122]. SA is a strong player in defense mechanisms alleviating adverse effects of abiotic stress in plants via SA-mediated regulation of plant metabolic processes. Therefore, plants respond to drought stress signals by activating defense systems through production of SA that acts as a signal transducer plant growth-regulating factor [123]. Several studies have underlined the role of SA in the induction of drought stress tolerance, however, the mechanisms behind SA-induced plant tolerance to abiotic stress, including drought circumstances, are still largely unknown [3]. SA induces the expression of a set of genes involved in plant abiotic stress tolerance such as non-expressor of pathogenesis-related genes 1 (NPR1), cytochrome P450, chaperones, heat shock proteins (HSPs), antioxidants, secondary metabolites [sinapyl alcohol dehydrogenase (SAD)], and mitogen-activated protein kinase (MAPK) regulation [124].

To elicit a drought-stress tolerance response, plants can be treated with exogenous SA via seed soaking, irrigation, or spraying [78, 124]. For example, exogenous application of SA to stressed-winter wheat plants increased drought tolerance, growth, and yield [125]. Moreover, exogenous application of SA and its derivatives, like acetyl-salicylic acid, through foliar spray or seed soaking, conferred drought stress tolerance to wheat (Triticum *aestivum*) [78], muskmelon (*Cucumis melo*) [126], tomato (*Lycopersicon esculentum L.*) and bean (*Phaseolus vulgaris L.*). Seed imbibition of SA or acetylsalicylic acid offers efficient stress tolerance and leads to expression of stress-related genes, which is consistent with the signaling role of these molecules. For example, both tomato and bean plants subjected to soil-drenching or grown from seeds treated in 0.1 to 0.5 mM SA or acetylsalicylic acid survived after drought stress while all the control plants died, showing that SA and its derivatives improve tolerance to drought stress. SA has been known to work through other plant hormones. For example, SA treatment in wheat resulted in an increase in ABA content, and in proline synthesis. When applied to drought-stressed plants, SA changed proline accumulation and ET production in mustard, altered proline content, plant growth, and physiological parameters in cowpea. SA supplementation improved photosynthetic activity, growth attributes and enhanced stomatal conductance in barley, which improved net CO_2_ assimilation rate and plant dry mass [127]. Exogenous SA has been shown to enhance enzymatic and nonenzymatic antioxidants defense and glyoxalase components systems, which decrease oxidative stress in drought-stressed mustard (*Brassica juncea* L.) [128]. Foliar application of SA enhanced the antioxidant defense system in the drought-tolerant *Z. mays*, and reduced membrane lipid peroxidation in *T. aestivum* [129].

Several SA producing beneficial rhizobacteria can colonize roots and cause them to be more resistant to drought stress, for example, inoculation of plants with SA producing strains of *Achromobacter xylosoxidans* and *Bacillus pumilus* enhanced drought stress. Khan et al. [130] showed that co-inoculation with two PGPR, *Planomicrobium chinense* strain P1 and *Bacillus cereus* strain P2 in combination with foliar SA treatment significantly increased accumulation of nutrients such as Cd, Pb, Ni, Cu, Co, and Zn in the rhizosphere, significantly increased leaf chlorophyll content, chlorophyll fluorescence, and carotenoids in the shoot compared to co-inoculation of PGPR P1 and P2 alone [130]. The combination of the PGPR consortium and SA treatment considerably induced other plant hormones, for example GA levels increase by 70%, and IAA production by 73%, but decreased the ABA content by 55%. In addition, co-application of PGPR and exogenous SA under drought stress significantly increased plant biomass, leaf water status, chlorophyll a and b, carotenoids synthesis, proteins biosynthesis, proline production, osmolytes production, defense-related system activities, and antioxidant enzymes production such as APX and CAT activities in the leaves [131]. *Pseudomonas chlororaphis* O6 induces production of SA through volatile organic compounds in *Arabidopsis*, which causes the plants to be tolerant to drought. Most PGPR induce drought stress through several processes, for example, delayed onset of drought symptoms, increased number of roots, root length, and root surface area were observed in wheat plants co-inoculated with *Bacillus* sp. and *Enterobacte*r sp. that produced both SA and IAA. In addition, drought stress causes leaf senescence, which aids in the remobilization of nutrients from the leaves, thus, the rest of the plant can benefit from the nutrients acquired during the leaf’s lifetime. This process can be carried out by the involvement of phytohormones, such as SA and JA. In perennial plants, leaf senescence is characterized by an increase in SA content up to 80% and a decrease in JA content by about 40% [132]. Hence, SA plays a role in drought stress regulation by inducing leaf senescence.

#### Role of volatile organic compounds

Plant volatile organic compounds (VOCs) are classified into small and heavier categories. Small VOCs include ET, and methanol, while heavier VOCs are compounds such as terpenes, methyl jasmonate, and methyl salicylate. The emission rates of volatile compounds are generally associated with the severity of stress [133]. Stress-induced VOCs act as priming signaling agents and have been implicated in systemic defense responses and in improving plant growth [33]. Furthermore, plants trigger their stress defense mechanisms when they perceive signals from drought-stressed neighboring plants. VOCs have become potential candidates for a rapid, non-invasive method of assessing drought-stressed plants [134]. PGPR can produce diverse groups of VOCs, such as alkenes, ketones, and alcohols, that are highly diffusible in the soil and plant cover [135]. Each bacterial strain produces distinct VOCs that play a significant role in the bacterial life cycle, have a direct effect on plant development, and indirectly control interactions between plants and other microorganisms [33]. Plants use certain VOCs as nutrition sources or as signaling molecules [136]. Some VOCs improve plant growth in a variety of ways, from stimulating seed germination to improving fruit production. Certain PGPR produce VOCs, which regulate the expression of numerous genes involved in expansion and rigidity of the plant cell wall. Several *Bacillus megaterium* strains can produce VOCs that augment the number of leaves and leaf surface area in *Arabidopsis*. *B. megaterium BOFC15* strain secretes a polyamine as a volatile molecule and promotes polyamine biosynthesis in *Arabidopsis*, resulting in an increase in plant biomass [8]. VOCs can enhance absorption of nutrients like iron and sulfur, and increase chlorophyll content and photosynthetic efficiency, which improves photosynthesis and carbohydrates synthesis [137].

Microbial VOCs may also promote plant tolerance to drought. Treatment of wheat with *Bacillus thuringiensis AZP2* enhanced drought stress tolerance: the plant biomass increased by up to 78% and there was a five-fold higher survival rate under severe drought stress because of significant reduction in volatile emissions [134]. *Bacillus subtilis GB03*, *B. amyloliquefaciens IN937a*, and *Enterobacter cloacae JM22* release several bioactive VOCs like 2,3-butanediol, and acetoin, which can act as signaling molecules to mediate growth of *Arabidopsis thaliana*. Colonization of *Arabidopsis* roots by *Pseudomonas chlororaphis O6* that produces volatile 2R, 3R-butanediol, induces tolerance to drought by reducing water loss through stomata closure [138]. Colonization of roots by P. *chlororaphis O6* deficient in 2R, 3R-butanediol did not lead to drought tolerance indicating that this VOC was required for induction drought stress tolerance. In sulfur deficient conditions PGPR can produce dimethyl-disulphide, a volatile sulfur source for plants. In addition, some VOCs produced by PGPR modulate plant hormone biosynthesis and homeostasis. For example, some VOCs play a role in auxin signaling biosynthesis and regulate cytokinin biosynthesis in *Arabidopsis*, and thus increase photosynthesis and plant development [90, 136]. *Bacillus subtilis* can produce VOCs which reduce ABA biosynthesis and subsequently maintain a normal photosynthesis apparatus because ABA prevents sugar buildup by interfering with photosynthesis. PGPR produced VOCs also play other roles in plant defense against phytopathogen, insects and herbivores [139].

#### Extracellular bacterial polysaccharides or exopolysaccharide

Beneficial bacterial biofilm contains several extracellular polysaccharides, such as exopolysaccharides (EPSs), lipopolysaccharides (LPSs), capsular polysaccharides (CPSs), and cyclic β -glucans [140]. These bacterial polysaccharides usually accumulate on cell surfaces and can contain a wide range of macromolecules that are beneficial to plant growth [138, 140]. Bacterial EPS are found in a large variety of complex structures that contain higher concentrations of proline, free amino acids, sugars, mono- and polysaccharides, and can have multiple functions in microbe-plant interactions under drought stress [141]. Some polysaccharides may have a greater water retention capacity than their mass and a little amount of the polysaccharide in biofilm can help maintain a hydrated microenvironment. Specifically, exopolysaccharide production is essential for the surface attachment [142], root colonization and cell aggregation [143], soil aggregation [64], the plant invasion process [143], nodule development [143], biofilm formation, and plant defense response [144], which confer protection against stress.

Water availability controls the production and consumption of bacterial polysaccharides, and thus indirectly influences soil structure [145]. Soil structure is an essential feature for sustainable agriculture because it affects a wide range of processes that influence crop output. Drought stress can alter soil physicochemical and microbial properties and subsequently agriculture yield. Rhizobacteria have evolved different adaptive strategies to counter drought stress and to survive in such a harsh environment. There is a considerable correlation between the amount of EPS generated by bacteria and their desiccation tolerance. Under drought stress, EPS protect the rhizosphere bacteria from desiccation by enhancing the availability of water, fertilizer and organic carbon to the colonized plant [145]. Roberson et al. [145] demonstrated that during desiccation conditions, *Pseudomonas* species increased EPS production, which improved the protection of the bacterial strain in soil. Moreover, the EPS released by soil microbes into soil as capsular polysaccharide and slime materials can be adsorbed by clay surfaces, thus forming a protective capsule around soil aggregates which can play an active signaling role during beneficial infections [146]. The addition of capsular components from *Azospirillum brasilense* Sp245 to a decapsulated solution of *A. brasilense* Sp245 increased drought resistance considerably [147]. EPS and other polysaccharide molecules produced by PGPR, are recognized to be important for promoting bacteria-plant interactions, which consequently improves plant survival and protection under stressful conditions [64]. Plants can influence production of EPS, in fact results from various experiments have shown that root exudates, primarily flavonoids, cause alterations in the PGPR-extracellular polysaccharide composition, thus influencing the PGPR–plant interaction [143]. EPS, which often represent 40–95% of the bacterial weight, are the most active elements of the extracellular soil organic matrix. Clay particles absorb the released EPS into soil and form a protective layer surrounding soil aggregates, protecting the plant from desiccation. For example, inoculation of sunflower rhizosphere with EPS-producing bacterial strain YAS34 under drought circumstances, showed a significant increase in the ratio of root-adhering soil to root tissue (RAS/RT) [148]. EPS create a water-retaining microenvironment that dries more slowly than the surrounding environment [149]. This can protect microorganisms and plant roots from desiccation. In fact, bacterial EPSs are highly hydrated compounds with 97% of water in polymer matrix, which impart protection against desiccation. Moreover, plants inoculated with EPS-producing bacteria showed better water and drought stress tolerance [150].

In addition, the bacterial EPS can generate a biofilm on the root surface, which could ultimately enhance soil structure, thereby ensuring plant development, and reducing the impact of drought stress on plants. For example, plants treated with EPS-producing *Azospirillum* strain displayed resistance to water stress through improvement in the soil structure and aggregation [150]. Inoculation of foxtail millet plants cultivated in semiarid regions in the northeast of China under drought conditions with *Pseudomonas fluorescens* DR7 strain showed higher levels of ACC deaminase and EPS-producing activity. This beneficial bacterium efficiently colonized the root adhering soil, enhanced soil moisture, and improved the RAS/RT ratio [151]. Bacterial EPS can enhance permeability by improving soil aggregation and maintaining a greater water potential surrounding the roots, resulting in increased nutrient absorption, plant development, and drought resistance [148]. Niu et al., [151] reported that several PGPR, including *Enterobacter hormaechei*, *Pseudomonas fluorescens*, and *Pseudomonas migulae* are drought-tolerant strains capable of producing EPS, which can enhance seed germination and seedling growth under drought conditions. Under drought stress, *Pseudomonas putida GAP-P45* strain producing EPS efficiently colonized the root adhering soil, increased soil aggregation, improved water and nutrient uptake, enhanced relative water content in the leaves, and plant biomass. Vurukonda et al., [14] reported that maize seed treated with the EPS-producing bacterial strains *Proteus penneri*, *Pseudomonas aeruginosa*, and *Alcaligenes faecalis* had higher soil moisture and relative water content, increased root and shoot length, leaf area, and plant biomass in drought stress conditions. The inoculated plants also showed an increase in protein, sugar and proline content, and decreased activities of antioxidant enzymes [152].

#### Metabolic and osmolyte regulation mediating drought stress alleviation

Plant drought tolerance is also controlled by the accumulation of various organic and inorganic secondary metabolites in the cytosol. These metabolites, also known as osmotic adjustments, are one of the most important strategies of plant adaptation to drought stress, and they as act compatible solutes that assist in maintaining plant metabolic activity [15]. Under drought stress, the accumulation of these osmotic adjustment compounds in the cytoplasm reduces the cell’s water potential and maintains the cell turgor, which promotes water absorption from the soil to the plant without interfering with normal metabolism [12]. Indeed, plant metabolic profiles do change in response to drought stress, implying that metabolic adjustments induce signal transduction and activation of stress tolerance in plants. Besides their role in signaling, these metabolites also act as antioxidants molecules and/or are involved in the defense system of plants [153]. These osmo-protectants can act as free radical scavengers, boosting antioxidant enzyme activity, preventing oxidation by eliminating excess ROS, and restoring cellular redox equilibrium [154]. Therefore, plants resort to a variety of adaptive methods that result in the accumulation of several important compatible solutes or osmolytes under stressful conditions. These osmo-protectants act as stress protectants in response to drought stress and they include compounds such as sucrose, maltose, trehalose, cellobiose, glutamate, polyhydric alcohols, glycine betaine, amino acids, proline, polyamines, organic acids, and water stress proteins like dehydrins [155].

Several studies indicate that beneficial microorganisms can generate compatible solutes to increase drought tolerance in plants. PGPR can synthesize osmolytes, which act synergistically with plant-produced osmolytes to promote plant development. Plants may take up these osmolytes from exogenous sources for example, under drought circumstances, chickpea (*Cicer arietinum*) inoculated with *Pseudomonas putida MTCC5279* strain exhibited modulation of membrane integrity, accumulation of osmolytes such as proline and glycine betaine, as well as the accumulation of antioxidant enzymes that can stimulate ROS scavenging [8]. Proline is one of the most investigated solutes because of its significant relevance to stress tolerance [12]. Proline is a low-molecular-weight organic amino acid that accumulates in plant leaves under low water potential. Under water deficit, ROS levels increase resulting in enhanced production of antioxidant and metabolites such as proline [156]. Proline accumulates as a solute in plants and plays an important role in different physiological processes, including protein solvation and structural stability, maintenance of membrane integrity, reduction-oxidation of lipid membranes, scavenging of reactive oxygen species, and buffering of cellular redox potential under stress conditions [157]. Proline accumulation under stress conditions has been linked to stress tolerance, and its concentration was higher in stress-tolerant plants than in stress-sensitive ones [12]. As a result, proline can operate as a signaling molecule to modify mitochondrial functions, impact cell proliferation or cell death, and stimulate specific gene expression, which are important for plant stress remediation and tolerance [158]. During post-drought recovery, proline also serves as a source of energy, carbon, and nitrogen [158]. The amount of proline in different plant tissues varies, and several factors influence its synthesis. For example, plant hormones may affect nutrient uptake; hence, they have a direct and indirect impact on proline content. Plants can use these nutrients to produce proline, for example nitrogen is important in proline metabolism. Proline accumulation in plants is the first response to water deficiency stress in order to protect cells from injury [12]. For instance, in water-stressed maize, progressive drought stress induced a significant accumulation of proline content [159]. Exogenous application of proline served as an osmo-protectant, and increased endogenous accumulation of free proline in drought-stressed petunia (*Petunia hybrida*), improving drought tolerance [160].

Inoculating plants with proline inducing PGPR increases proline concentrations in plants. For example, maize (*Zea mays* L.) inoculated with *Pseudomonas fluorescens* under drought stress showed a significant increase in proline level [161]. In another study, maize plants inoculated with *Pseudomonas putida GAP-P45* strain showed an accumulation of proline and soluble sugars, which enhanced relative water content, leaf water potential, and plant biomass under drought stress compared to non-treated plants. Similarly, to cope with the drought stress, tomato (*Lycopersicon esculentum Mill*) plants inoculated with phosphate-solubilizing *Bacillus polymyxa* released an excessive amount of proline, which improved the physiological and biochemical properties of the host plant [49]. When *Lavandula dentata* plants under drought stress were treated with IAA producing *B.thuringiensis*, they showed an enhanced proline and K-accumulation in shoots, which improved nutritional, physiological, and metabolic activities, depressed stomatal conductance, and decreased glutathione reductase and ascorbate peroxidase activity, all of which are stress response processes in plants [56]. The increased proline content in rice (*Oryza sativa* L.) inoculated with PGPR consortia resulted in higher plant tolerance to water stress, suggesting the key role of proline as an osmoregulatory solute in regulating cell water status and protecting membranes and proteins from stress in PGPR treated plants. Another example showed that a set of beneficial strains known as BBS, which included *Bacillus cereus AR156*, *B. subtilis SM21* and *B.serratia sp. XY21* improved drought stress tolerance in cucumber plants through increased concentration of proline and photosynthetic pigments, root recovery, and reduction of downregulation of drought-encoded genes as well as the small and large Rubisco subunits. Proline is the most important amino acid for polyamine production in diverse plant species and plant growth is influenced by polyamines synthesis [3]. Cationic polyamines can form associations with anionic membrane components, such as phospholipids, and subsequently protect the lipid bilayer from the damaging effects of stress [162].

Trehalose is also a highly stable nonreducing disaccharide. High levels of trehalose can play a significant role in plant growth and protection against drought stress [82]. The heterologous expression of trehalose-producing genes in *Escherichia coli* or *Saccharomyces cerevisiae* improved drought tolerance in plants [163]. For example, the over-expression of the trehalose-6-phosphate synthase gene OsTPS1 enhanced drought resistance to rice. Treatment of plants with PGPR that overproduce trehalose can be an efficient strategy for coping with drought stress [164]. For example, plant productivity, yield, and drought resistance were all improved when maize plants were exposed to the PGPR *Azospirillum brasilense* that had been engineered to overproduce trehalose [164].

#### Antioxidant defense system

Drought stress enhances photorespiration and disrupts photosynthesis and cell homeostasis, which causes the release of reactive oxygen species (ROS) [3, 12, 15]. ROS include singlet oxygen (^1^O_2_), hydroxide (OH^−^), hydrogen peroxide (H_2_O_2_), hydroxyl radicals (^•^OH), superoxide anion radicals (O_2_^•−^), perhydroxyl radical (HO_2_^•^), and free and alkoxy radicals (RO) 14,165]. ROS are usually generated at a low level in many organelles, such as chloroplasts, mitochondria, and peroxisomes, during optimal growth conditions. However, one of the inevitable consequences of drought stress is a dramatic increase in the rate of ROS production in various cellular compartments [15]. ROS are toxic and highly reactive and cause damage to DNA, proteins, lipids and carbohydrates leading to oxidative stress, general oxidative damage to cells, impairment of normal plant cell functioning, and eventually programmed cell death (PCD) [165]. In recent decades, research has shown that ROS are involved in many signaling transduction pathways in response to stress perception, including photosynthetic regulation, hormonal activity, and plant growth, to the ultimate triggering of plant cell defensive reactions and drought stress responses [166]. Drought stress affects ROS photoproduction, which occurs when the amount of absorbed light energy exceeds that utilized for CO2 assimilation [167]. In fact, photosystem I (PSI) and photosystem II (PSII) in chloroplast thylakoids are the primary source of ROS production, and most of ROS generation in chloroplasts is associated with an over-reduction of the electron transport chain [168]. Drought causes a surge in the accumulation of these different intracellular ROS, which leads to a drastic imbalance in cellular functions such as carbohydrate degradation, chlorophyll damage, protein and lipid oxidation, nucleic acid fragmentation, and membrane lipids deterioration, which can negatively impact carbohydrate metabolism, respiration, photosynthesis, as well as limit growth of drought-stressed plants [169]. Typically, the plant scavenges and detoxifies the injurious ROS through an antioxidant defense mechanism, which regulates the intracellular ROS level, ensures an equilibrium between the production and elimination of ROS and defines the redox status of the cell [15]. Thus, maintaining the balance between ROS production and ROS detoxification is essential to plant cell viability [134]. As a result, the balance between ROS generation and antioxidant enzyme activities determines whether oxidative signaling or damage will occur. The antioxidant regulation network adjusts the equilibrium level of ROS for signaling and defense purposes through regulating the production rates of ROS and ROS scavenging enzymes [170].Under drought conditions, plants suffer from oxidative damage, causing an imbalance between the accumulation of reactive oxygen species and the ability of the plants’ cellular antioxidant system to control ROS levels [41].

**Fig. 2:**
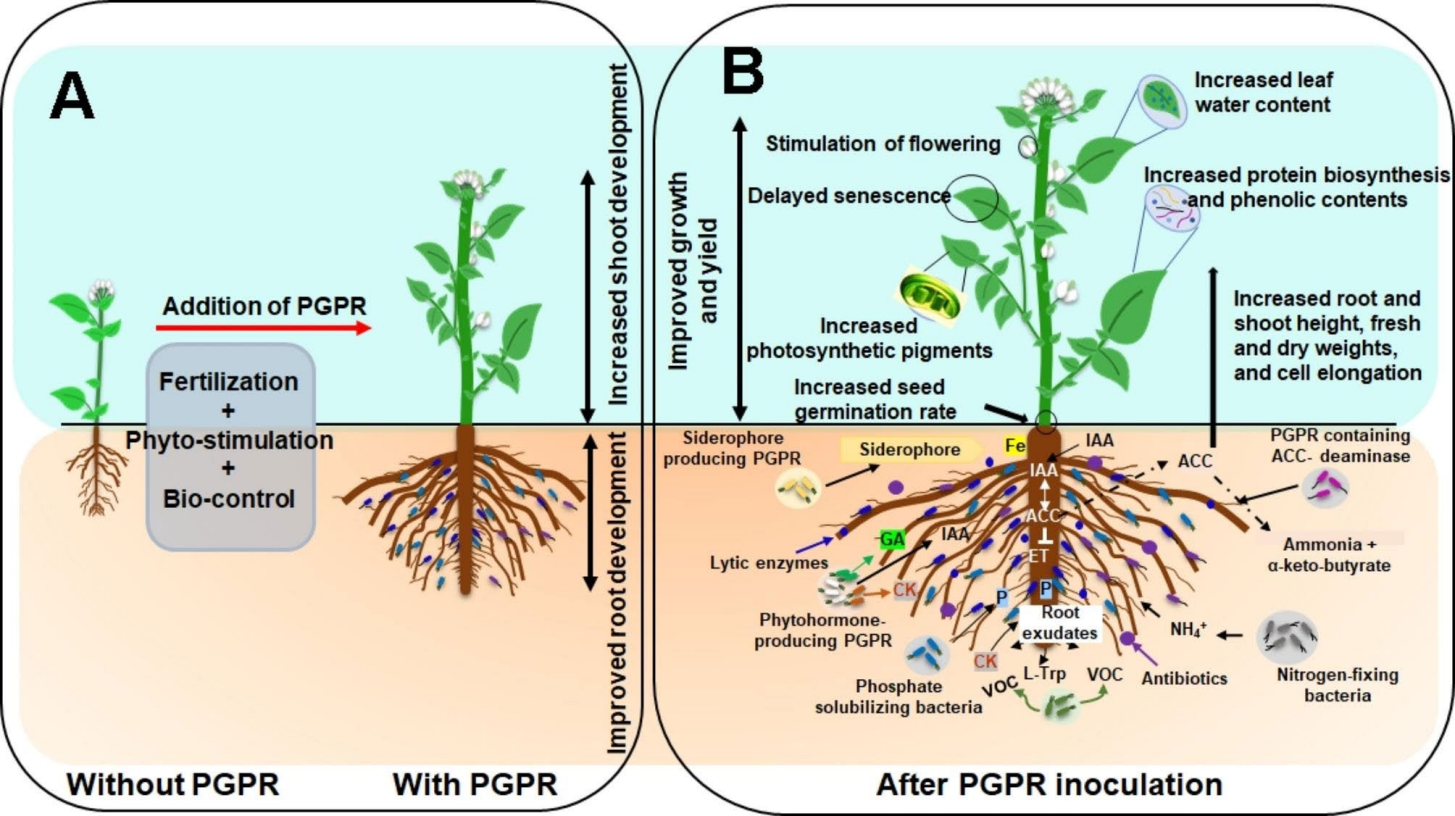
A schematic overview of the processes used by plant growth promoting rhizobacteria to alleviate drought stress and promote plant growth. **A**. Plant growth promoting rhizoba*cteria* improve general traits of plant growth. Inoculation with PGPR increased the growth rate and general developmental of plants compared to the non-inoculated control (on the left). PGPR enhance root system architecture, root and shoot growth and weight, height, and early plant flowering. **B**. Functional processes used by plant growth promoting rhizobacteria to promote plant growth: PGPR colonization of plant roots enlarges root architecture and enhances, nutrient and water uptake, nitrogen fixation, phytohormone production, enzyme production, photosynthetic activity and other processes. PGPR: Plant Growth Promoting Rhizobacteria; ACC: 1- AminoCyclopropane-1-Carboxylate; CK; Cytokinin; ET: Ethylene; Fe; Iron; GA: gibberellic acid; IAA: indole-3-acetic acid; N: Nitrogen; NH3: ammonia; NH_4_^+^: ammonium; P: Phosphate; L-Trp: L-tryptophan; VOC: volatile organic compound

**Fig. 3:**
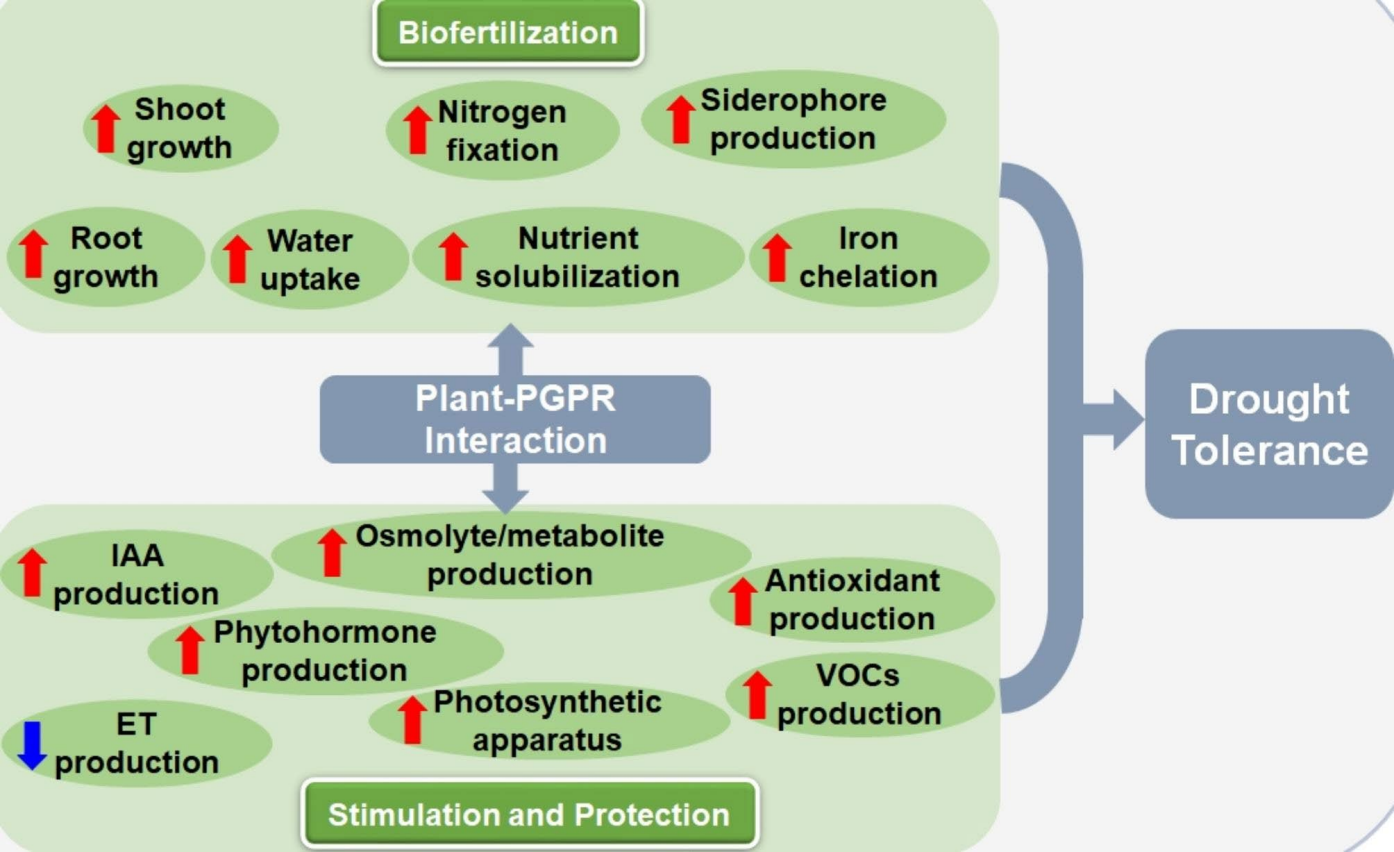
Thematic diagram showing mechanisms adopted by plant growth-promoting rhizobacteria to induce drought tolerance in plants. Plant growth-promoting rhizobacteria (PGPR) interact with host plants through several mechanisms to promote drought tolerance in the host. These mechanisms fall into two broad categories: biofertilization, and stimulation and protection. PGPR: Plant Growth Promoting Rhizobacteria; ET: Ethylene; IAA: indole-3-acetic acid; Vocs: volatile organic compounds

Plants have both enzymatic and non-enzymatic antioxidant defense systems that act to scavenge ROS [3]. Enzymatic antioxidants include metalloenzyme, SOD, ascorbate peroxidase (APX), peroxidase (POD), CAT, glutathione peroxidase (GPx), glutathione reductase (GR), and polyphenol oxidase [171]. SOD can dismutase O_2_^−^ into H_2_O_2_, which is then scavenged by POD in different plant compartments such as chloroplast, mitochondria, and peroxisome [12]. CAT is a key enzyme that also scavenges H_2_O_2_ from the mitochondrion and microbody, therefore reducing the negative impact of oxidative stress. Additionally, APX, dehydro-ascorbate reductase, monodehydroascorbate reductase, and GR are involved in the ascorbate-glutathione cycle that permits the scavenging of O_2_^•−^ in the chloroplast, mitochondria, peroxisome, and cytosol [172]. Whereas the non-enzymatic antioxidants include phenolic compounds, flavanones, alkaloids, carotenoids, ascorbic acid, cysteine, reduced glutathione, and α-tocopherols antioxidants [173]. Plants’ carotenoids are an important component of the plant antioxidant defense system, which scavenges ^1^O_2_ and lipid peroxyl-radicals, as well as inhibits lipid peroxidation and superoxide production under dehydration stress [174]. β-Carotene binds to the core complexes of photosystems I and II in the chloroplasts of plants, acting as an antioxidant that quenches triplet chlorophyll, prevents the production of ^1^O_2_, and protects plants from oxidative damage caused by ROS [174, 175]. PGPR have been shown to alleviate drought stress by detoxifying ROS. Under severe drought conditions, inoculating lettuce (*Lactuca stiva* L.) with *Pseudomonas mendocina* showed increased activity of CAT, which scavenges H_2_O_2_ to help plants cope with the oxidative stress, suggesting that PGPR inoculant can be used as an eco-friendly tool to alleviate the oxidative damage caused by drought. Similarly, CAT activity was observed to increase in mung bean (*Vigna radiata*) plants inoculated with a *Pseudomonas aeruginosa* strain under drought conditions [176]. Likewise, under water stress, basil plants (*Ocimum basilicum* L.) treated with *Pseudomonas sp*., significantly increased the activity of CAT, GPx, and APX enzymes and plants treated with a microbial consortia consisting of *Pseudomonades sp*., *Bacillus lentus*, and *Azospirillum brasilense* showed similar results [177]. Inoculating basil plants with *Pseudomonades sp*., increased antioxidant activities and photosynthetic pigments. In addition, Nautiyal et al., [178], showed that the PGPR *Bacillus lentimorbus* boosted the antioxidant capacity in lettuce, spinach, and carrot plants, which improved the growth and development of these inoculated plants. Maize seedlings inoculated with *Bacillus amyloliquefaciens* SQR9 strain exhibited enhanced total soluble sugar content, which led to lower cell destruction, higher POD and CAT activity, and glutathione content for scavenging ROS [179]. *Bacillus thuringiensis* increased growth and drought resistance of *Lavandula dentata* by raising K content, boosting IAA and shoot proline production, and decreasing APX and GR activities, which reduced cellular oxidative damage [56]. Therefore, PGPR can use similar adaptive mechanisms to alleviate the drought stress and promote plant growth (Figs. 2 and 3).

Despite the strides that have been made in understanding the potential benefits of PGPR to plants, more studies still need to be carried out to unravel the complexities of the interactions between different PGPR and plant species. Better understanding of the role of PGPR in enhancing plant growth and tolerance to abiotic stresses will lead to their widespread adoption in agriculture production systems. Given the great variability in the PGPR capabilities and crop needs, there is need to come up with combinations PGPR consortia and other microorganisms such as arbuscular mycorrhizal fungi that are adapted to specific crop needs and environmental conditions.

## Conclusion

Drought is a multidimensional common stress factor that negatively impacts plant growth, development, and metabolism. Thus, drought stress is a complex phenomenon adversely affecting plants at multiple levels. Plants have evolved a variety of tolerance strategies at molecular, developmental, physiological, morphological, and biochemical levels to cope with drought stress. These drought stress responses can be direct or indirect, and include regulation of water content, stomatal movements, leaf senescence, osmolytes adjustment, antioxidant metabolism, photosynthetic activity, and phytohormonal production. Different groups of beneficial bacteria that colonize plant roots promote plant growth and development, and stress tolerance through a variety of strategies. In their role as eco-friendly bio-fertilizers, PGPR can alleviate the detrimental effects of drought on plants by enhancing growth under water deficit through different processes. PGPR can promote plant growth and alleviate drought stress using the same strategies. Drought-stressed plants inoculated with PGPR display several adaptive responses to maintain water potential in the tissues such as osmotic adjustment, production of osmoprotectants and growth regulators, as well as increased antioxidant activity. Here we provided an overview of the current knowledge on the beneficial interactions between plants and PGPR, and the underlying mechanisms that mitigate the impact of drought stress. The application of PGPR can be an effective tool to induce drought tolerance and sustain productivity in drought-stressed plants.

## Data Availability

All data generated and/or analyzed during this study are available from the corresponding author on reasonable request. No sequencing, genomic, or phylogenetic data were generated during this study.

## List of abbreviations

^•^OH: Hydroxyl radicals
^1^O_2_: Singlet oxygen
ABA: Abscisic acid
ACC: 1-AminoCyclopropane-1-Carboxylate
ACS: 1-Aminocyclopropane-1-carboxylate synthase
APX: Ascorbate peroxidase
Ca: Calcium
CAT: Catalase
CK: Cytokinin
CO_2_: Carbon dioxide
CPSs: Capsular polysaccharides
DRE: Dehydration-responsive element
DREB1A: Dehydration-responsive element-binding protein 1 A
EPS: Extracellular polysaccharides
ET: Ethylene
GB: Glycine-betaine
GPX: Glutathione peroxidase
GR: Glutathione reductase
H_2_O_2_: Hydrogen peroxide
HO_2_^•^: Perhydroxyl radical
HSPs: Heat shock proteins
IAA: Indole-3-acetic acid
IAM: Indole-3-acetamide
IAN: Indole-3-acetonitrile
IPA: Indole-3-pyruvic acid
ipdC: Indole-3-pyruvate decarboxylase
ISR: Induced systemic resistance
JA: Jasmonic acid
K: Potassium
LPSs: Lipopolysaccharides
MAPK: Mitogen-activated protein kinase
Mg: Magnesium
NCED: 9-cis-epoxycarotenoid dioxygenase
NH3: Ammonia
NH_4_^+^: Ammonium
NPR1: Non-expressor of pathogenesis-related genes 1
O_2_^•−^: Superoxide anion radicals
OH^−^: Hydroxide
P: Phosphate
PCD: Programmed cell death
PGPR: Plant Growth Promoting Rhizobacteria
POD: Peroxidase
PS: Photosystem
RAS: Root-adhering soil
RO: Free and alkoxy radicals
ROS: Reactive oxygen species
RT: Root tissue ratio
Rubisco: Ribulose-1,5-bisphosphate carboxylase
S-AdoMet: S-adenosylmethionine
SA: Salicylic acid
SAD: Sinapyl alcohol dehydrogenase
SAR: Systemic acquired resistance
Snf: Sucrose non fermenting
SnRK2: Sucrose non fermenting 1-related protein kinase 2s
SOD: Superoxide dismutase
VOC: Volatile organic compound
